# Structural equation modeling with latent variables for longitudinal blood pressure traits using general pedigrees

**DOI:** 10.1186/s12919-016-0047-4

**Published:** 2016-10-18

**Authors:** Yeunjoo E. Song, Nathan J. Morris, Catherine M. Stein

**Affiliations:** 1 Department of Epidemiology and Biostatistics, Case Western Reserve University, Cleveland, OH 44106 USA; 2 Center for Clinical Investigation, Case Western Reserve University, Cleveland, OH 44106 USA; 3 Center for Proteomics and Bioinformatics, Case Western Reserve University, Cleveland, OH 44106 USA

## Abstract

Structural equation modeling (SEM) has been used in a wide range of applied sciences including genetic analysis. The recently developed R package, *strum,* implements a framework for SEM for general pedigree data. We explored different SEM techniques using *strum* to analyze the multivariate longitudinal data and to ultimately test the association of genotypes on blood pressure traits. The quantitative blood pressure (BP) traits, systolic BP (SBP) and diastolic BP (DBP) were analyzed as the main traits of interest with age, sex, and smoking status as covariates. The single nucleotide polymorphism (SNP) genotype information from genome-wide association studies (GWAS) data was used for the test of association. The adjustment for hypertension treatment effect was done by the censored regression approach. Two different longitudinal data models, autoregressive model and latent growth curve model, were used to fit the longitudinal BP traits. The test of association for SNP was done using a novel score test within the SEM framework of *strum*. We found the 10 SNPs within the GWAS suggestive *P* value level, and among those 10, the most significant top 3 SNPs agreed in rank in both analysis models. The general SEM framework in *strum* is very useful to model and test for the association with massive genotype data and complex systems of multiple phenotypes with general pedigree data.

## Background

Structural equation modeling (SEM) has been used in a wide range of applied sciences as well as in genetic analysis [1, 2], particularly for longitudinal data analysis [3, 4]. SEM is a general and powerful approach to account for measurement error and causal pathways by estimating the parameters for a system of simultaneous equations [5, 6]. The R package *strum* was recently developed [7], implementing the framework for SEM for general pedigrees described in Morris et al. [8]. It includes both fitting and simulation of a broad range of latent measurement models and structural equation models with covariates, allowing for a wide variety of models including latent growth curve models. It can handle multilevel models, polygenic random effects and linkage random effects. Traditional structural equation models and confirmatory factor analysis may also be performed.

The Genetic Analysis Workshop 19 family data set includes the longitudinal multivariate blood pressure traits. This complexity of traits in this data set provides a good opportunity to evaluate the flexibility and applicability of the *strum* package for modeling in family data. This paper explores the 2 different SEM techniques using *strum* to analyze the multivariate longitudinal data and, ultimately, to test the association of genotype to blood pressure (BP) traits, looking for a set of single-nucleotide polymorphisms (SNPs) that came up as significant in both analysis models.

## Methods

### Data

We analyzed the real family data set, which consists of 1389 individuals from 20 families with 27 to 107 members. The detailed description of the data can be found in Almasy et al. [9]. The quantitative BP traits, systolic BP (SBP) and diastolic BP (DBP) were analyzed as the main traits of interest. We included age, sex, and smoking status as covariates. For both methods, we only included the first 3 visits into our analysis as more than 80 % of data were missing for the 4th visit, and there were 10 families with completely missing data for the 4th visit. The genotype information from genome-wide association studies (GWAS) data was used, which included 472,060 SNPs in total. After removing the SNPs with no variation in the data set or with no score test results, the remaining 460,359 SNPs were tested for association with the main traits by coding additively as 0, 1, or 2 based on the minor allele count.

Before being included in any structural equation analysis, the BP trait value at each visit was adjusted for the effect of hypertension medication as done by other researchers, to reduce the bias in the estimated effect of interest and the loss in statistical power [10]. We followed the censored regression approach of Konigorski et al. [11]. The difference between the observed and fitted BP for the untreated individuals and the difference between the adjusted and fitted BP for the treated individuals are used as our main BP trait values (denoted as rSBP and rDBP).

### Analysis

**Fig. 1:**
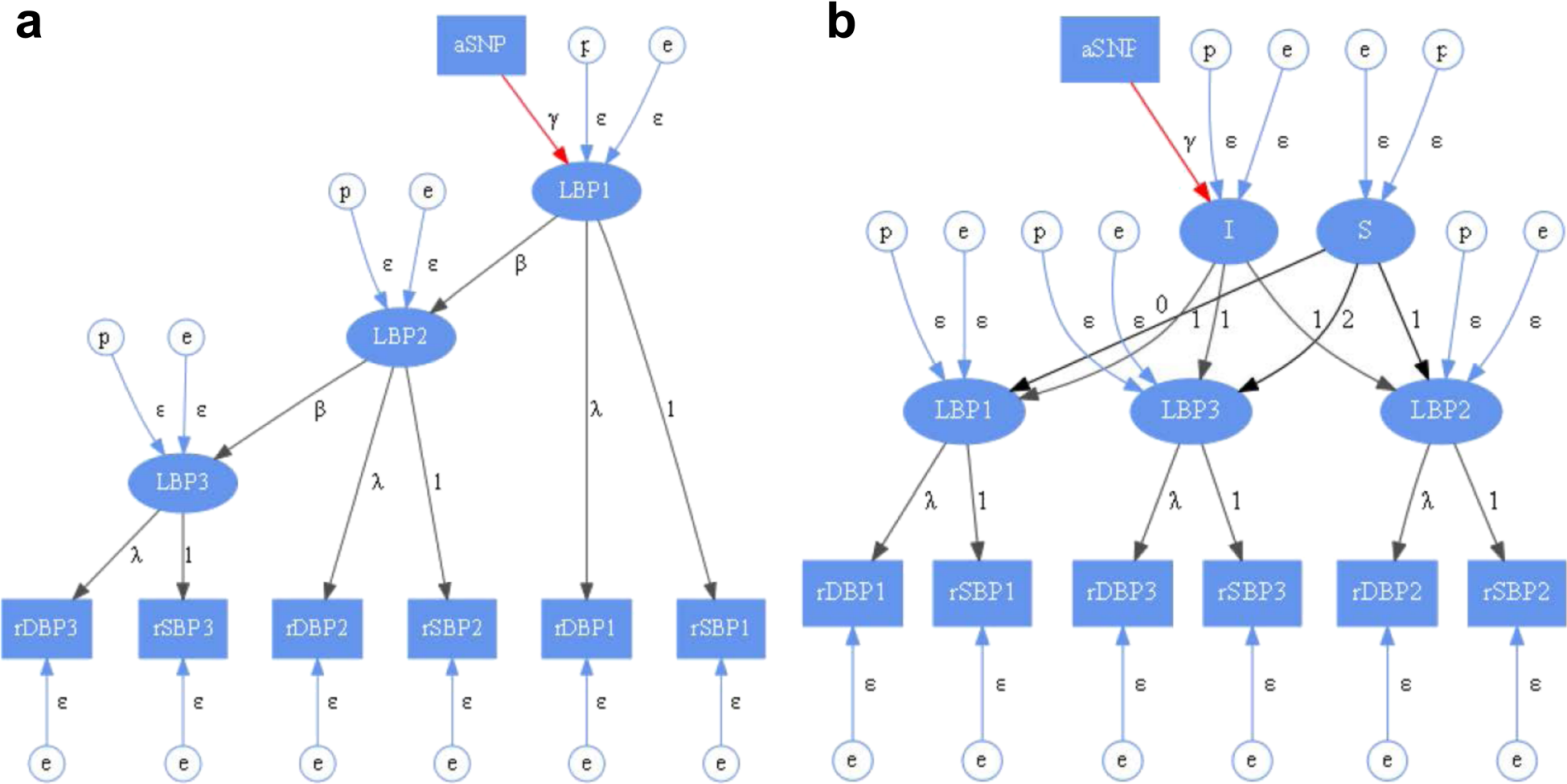
Analysis models. The graphical representations of analysis models with latent variable for longitudinal blood pressures are shown for: **a** an autoregressive model and **b** a latent growth curve model. Variables rSBP and rDBP are the main trait values corrected for the use of hypertension medication. LBP is a latent variable, and aSNP is a SNP tested. Nodes marked with: “p” are polygenic effects, and “e” are random effects. I is the intercept and S is the slope. Note that the coefficient to test the SNP effect on blood pressure traits is colored in red

We included 2 different types of longitudinal data modeling approaches in this study: autoregressive (AR) model and latent growth curve (LG) model. In both models, the observed values of SBP and DBP at each time point are assumed to include measurement errors. In each time point, it is assumed that there is a latent variable that affects both SBP and DBP in both models. However, the relationship among the latent variables at different time points and the SNP effect on the underlying latent variables are differently modeled in each analysis model. Therefore, the number of parameters estimated in the correlation structure is different in each model. The visual presentations of the 2 models are shown in Fig. 1. The pedigree relationship is incorporated in the model by including and simultaneously estimating the polygenic effect denoted as *circled p* in Fig. 1.

#### Model 1: autoregressive model with measurement error

This is a first-order AR model with measurement error. In this model, the latent variable at t(n) is a function of the latent variable at t(n-1) and not any variable before, so the true underlying latent variable has an AR structure. The SNP effect is modeled directly on the latent variable in the first time point and indirectly to other time points.

#### Model 2: latent growth curve model with latent slope and intercept

This is a method to study growth (or change) over time. In this model, the latent variables at different time points share a common intercept with different slopes. The model includes the SNP effects directly on the intercept, so the SNP effects all time points equally. This approach models individual change process as function of latent intercept and slope factors.

Based on the original *strum* framework, we developed a new score test. This method is a computationally rapid test of association with many SNPs in GWAS data (manuscript in preparation). In this new score test, we first assessed the null model fit without any SNPs in the model to confirm the appropriateness of the model for the data. For each model, we ran the analysis 3 times to make sure the results were fully converged. Then, one at a time, each SNP was tested for association with the BP traits.

## Results

**Fig. 2:**
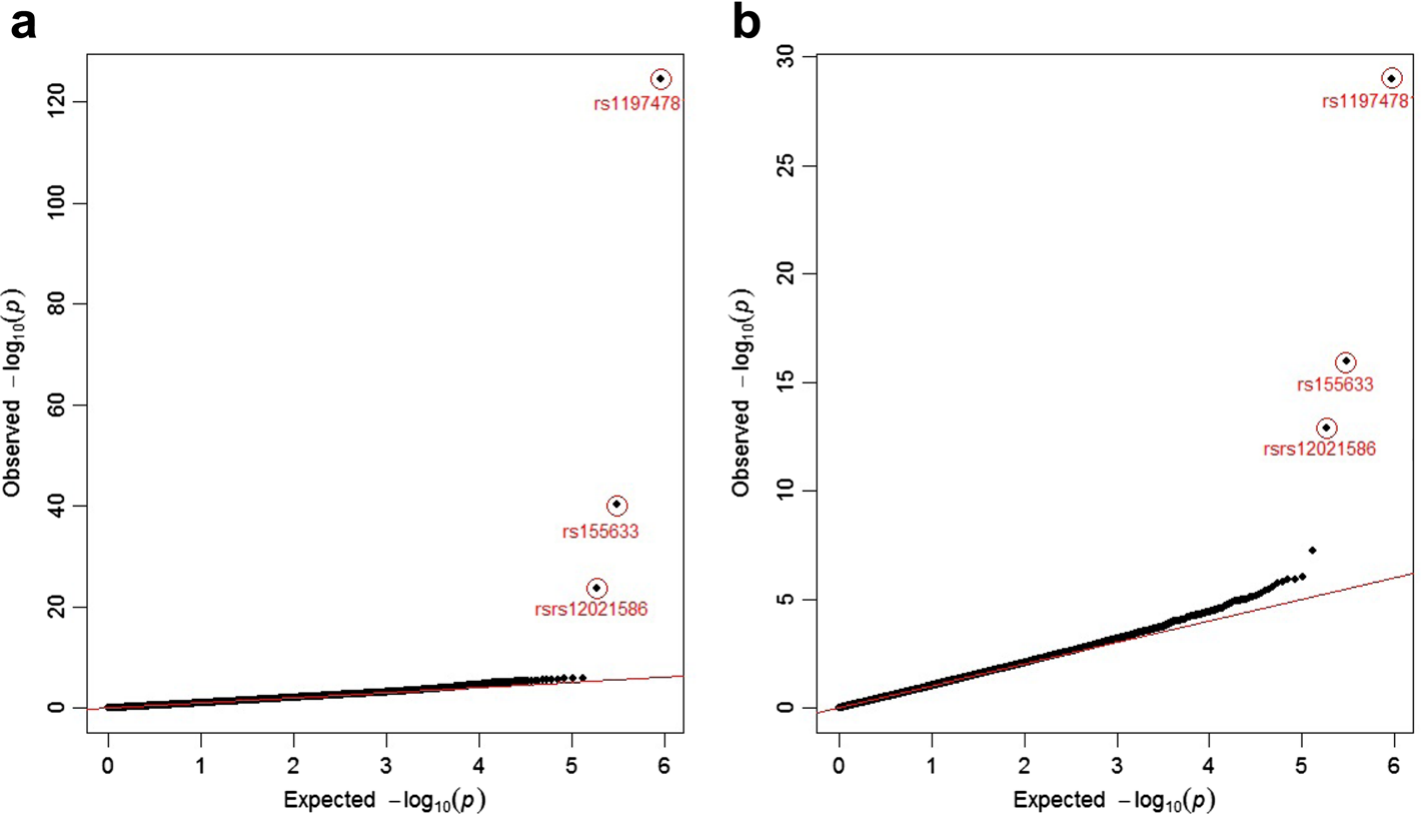
Quantile–quantile (Q-Q) plots of *P* values from genome-wide association study. The Q-Q plots of the observed and expected *P* values of GWAS result are shown for **a** an autoregressive model and **b** a latent growth curve model

The overall results of the association tests for all SNPs are shown in Fig. 2 as the quantile–quantile (Q-Q) plots of the observed and expected *P* values for 2 analysis models. The genomic inflation factors were 1.01 and 1.02 for each model. Even though the SNP effect was modeled differently, the ranking of *P* values from both models was very close (correlation = 0.9). Out of total 460,359 SNPs tested, there were 26 SNPs for the AR model and 18 SNPs for the LG model with suggestive evidence of association (ie, *P* value < 1.0e-5). Among those, 10 SNPs were the same from both models, and 4 SNPs on chromosome 1 are located close to each other indicating that they are in high linkage disequilibrium (LD). Table 1 shows the characteristics and *P* values. Interestingly, the most significant top 3 SNPs from both models agreed in rank as shown in Fig. 2.

**Table 1:** SNPs associated with SBP and DBP in both analysis models

Ch	SNP	BP	Known gene	Al	MAF	AR *P* value	LG *P* value
1	rs155633	29928916		G/*T*	0.0232	5.510E-41	1.015E-16
1	rs12021586	35629454	PSMB2	G/*T*	0.0102	2.057E-24	1.223E-13
1	rs4453019	196101652		C/*T*	0.1851	4.190E-06	3.911E-06
1	rs11584379	196114488		T/*G*	0.1448	3.916E-06	3.548E-06
1	rs6696438	196115362		C/*T*	0.1476	2.153E-06	1.910E-06
1	rs16839516	196135852		G/*A*	0.1382	2.389E-06	1.660E-06
7	rs11974781	147348978	CNTNAP2	G/*T*	0.0208	2.31E-125	1.011E-29
11	rs10792447	64824500	CDC42BPG	T/*C*	0.4115	7.450E-06	2.640E-06
13	rs4143295	107787911	FAM155A	T/*G*	0.1314	2.059E-06	8.258E-06
17	rs3760323	35433147	SLFN12	C/*T*	0.1208	7.443E-06	7.455E-06

Information on 10 SNPs from both analysis models with *P* value < 1.0e-5

*Al* major/minor alleles, *BP* base position, *Ch* chromosome, *MAF* minor allele frequency

## Discussion

There have been several recent genetic studies on BP traits [12, 13]. In most studies, 2 BP traits, SBP and DBP, are analyzed separately or they are summarized into 1 value. In addition, the longitudinal values are also summarized into a value. In our study, we report the SNPs associated with the latent variable for both BP traits longitudinally. Therefore, our results and the results from the association test on the summarized BP trait may not be easily comparable, and our results provide different GWAS findings. However, the differences and agreements of the results from ours and from the analysis done in each time points separately might give another interesting and useful insight into the relation between BP traits and genotypes.

The unbalanced missing rates in each time points with the longitudinal data were a limitation with this study. We were only able to include the first 3 visits into our analysis since there were 10 families with the completely missing data for the 4th visit which would have reduced the effective sample size to 10 from the original 20 families.

Similar results were found from 2 different analysis models, but there were differences in magnitude of *P* values for the top hits. This might be a result of the differences in the number of parameters in the models. Also, the highly significant *P* values for the same top 3 SNPs from both models might be a result of the low minor allele frequency (MAF). Upon further examination, we found the minor allele was not present in many families, reducing the effective sample size. The effect of MAF on type I error in family data using this approach needs to be investigated.

## Conclusions

The initial version of the novel score test we have developed is computationally efficient enough for genome wide analysis, but its statistical properties need to be more fully evaluated. Among the results from 2 analysis models, that is, the AR model and LG model, we found the 10 SNPs within the GWAS suggestive *P* value level, and among those 10, the most significant top 3 SNPs agreed in rank in both analysis models. The similar results from both models provide more confidence on the results. The general SEM framework in *strum* is very useful to model and test for the association with massive genotype data and complex systems of multiple phenotypes with general pedigree data.
